# Service-Life Prediction of Core Inserts Under High-Volume Plastic Injection Molding Conditions

**DOI:** 10.3390/ma19153307

**Published:** 2026-08-04

**Authors:** Hamza El Fahime, Mohammed Radouani, Benaissa El Fahime

**Affiliations:** Laboratory of Mechanics, Mechatronics and Control—(L2MC), ENSAM, Moulay Ismail University, Meknes 50050, Morocco; h.elfahime@edu.umi.ac.ma (H.E.F.); m.radouani@ensam-umi.ac.ma (M.R.)

**Keywords:** plastic injection molds, high-volume production, AISI H11 core inserts, failure root-cause analysis, computer-aided engineering tools, TMF-creep lifetime prediction

## Abstract

**Highlights:**

**What are the main findings?**

- The insert failure initially appeared to be caused by a possible overload. However, the breakage root cause analysis indicates a cumulative damage mechanism driven by exposure to continuous injection molding production boundary conditions.
- The study established a direct correspondence between the observed rupture location and areas of stress concentration simulated under cyclic molding conditions, evaluating the structural response of the mold’s core insert.
- A clear prediction gap was identified when using a classical isothermal low-cycle fatigue model, indicating that conventional LCF assessment alone may be insufficient to explain premature failure.

**What are the implications of the main findings?**

- Core insert durability can be evaluated using a coupled process, structure, and damage approach when static resistance verification alone is not sufficient.
- Progressive damage is driven by the combined effects of cyclic pressure, conduction and convection, and assembly constraints requiring a multiphysics assessment.
- CAE tools serve as a bridge between production conditions and structural stress evaluation.
- This direction provides a foundation for digital-twin monitoring and AI-assisted diagnostic frameworks capable of failure detection, root-cause identification, and preventive action for condition-based maintenance support in molding systems.

**Abstract:**

High-volume injection molding systems for plastic part production operate under severe thermo-mechanical loading repeated at high production rates. Therefore, premature breakage of hot-work tool steel core inserts directly interrupts production continuity and creates a recurrent maintenance problem. To address this failure mode, this study collected production inputs and failure evidence and established a reliable interpretation by linking computer-aided engineering simulations of the plastic injection molding process, structural FEA of the insert response, and cumulative fatigue assessment using a TMF-creep damage model. This integrated approach provides a comprehensive framework for failure root-cause identification and service-life prediction and improving insert durability in mass-manufacturing environments.

## 1. Introduction

Plastic injection molding is a high-volume manufacturing process used to produce polymer components with complex geometries and repeatable dimensional accuracy. During repeated production cycles, the mold is not only a geometric tool used to shape the polymer but also a mechanical and thermal system exposed to repeated loading [1].

The reliability of mold components is therefore essential, especially when production is continuous and a localized insert failure can interrupt the process, increase maintenance costs, and affect product quality [2]. Core inserts are among the most sensitive mold components because they are positioned in regions where polymer pressure, transient heat transfer, mechanical support, and contact constraints interact. During filling and packing, the molten polymer applies non-uniform pressure to the cavity and insert surfaces. During cooling and solidification, transient thermal exposure causes a local response. These repeated actions create a coupled thermo-mechanical environment in which the insert response cannot be understood from global machine settings alone [3]. Previous CAE-based studies have shown that injection-molding simulation can provide non-uniform melt-pressure fields that can be transferred to core stress analysis for fatigue-life evaluation, as demonstrated by Kim and Lee [4]. This supports the use of a process-to-structure approach, where the molding process first defines pressure and temperature fields, and structural simulation then evaluates the local response of the insert. However, identifying a stress concentration is not sufficient by itself to explain premature breakage, because service life also depends on cyclic loading, thermal exposure, and damage accumulation.

AISI H11 steel is widely used for thermally and mechanically loaded tooling applications because of its hot-work performance. Under cyclic molding conditions, however, insert durability cannot be described only by classical static assessment or baseline low-cycle fatigue models [5]. The material response is affected by numerous excitations interfering with the damage evolution [6]. The present work therefore considers the interaction between creep, thermal fatigue, and cyclic loading, which is fundamental for accurate insert service-life prediction [7]. This need is supported by experimental fatigue studies on AISI H11/H13 test specimens, thermal-fatigue investigations, and high-temperature damage-modeling approaches for AISI H11/X38CrMoV5/DIN 1.2343-type steels [8].

The scope of this paper is therefore focused on an industrial AISI H11 core insert failure case under high-volume injection molding conditions, where the mold produces 16 parts per cycle with a cycle time of 15.2 s. The study addresses the gap of establishing a reproducible root-cause identification approach. Specifically, it links visual failure evidence, process measurements, and structural finite element analysis response with LCF and coupled TMF-creep damage life assessments [9].

Accordingly, this case study explores how an observed core insert breakage can be evaluated through a structured process to structure to damage life-assessment workflow. The purpose is to determine whether the failure root-cause hypothesis is consistent with the results of the applied analysis models.

## 2. Materials and Methods

**Figure 1:**
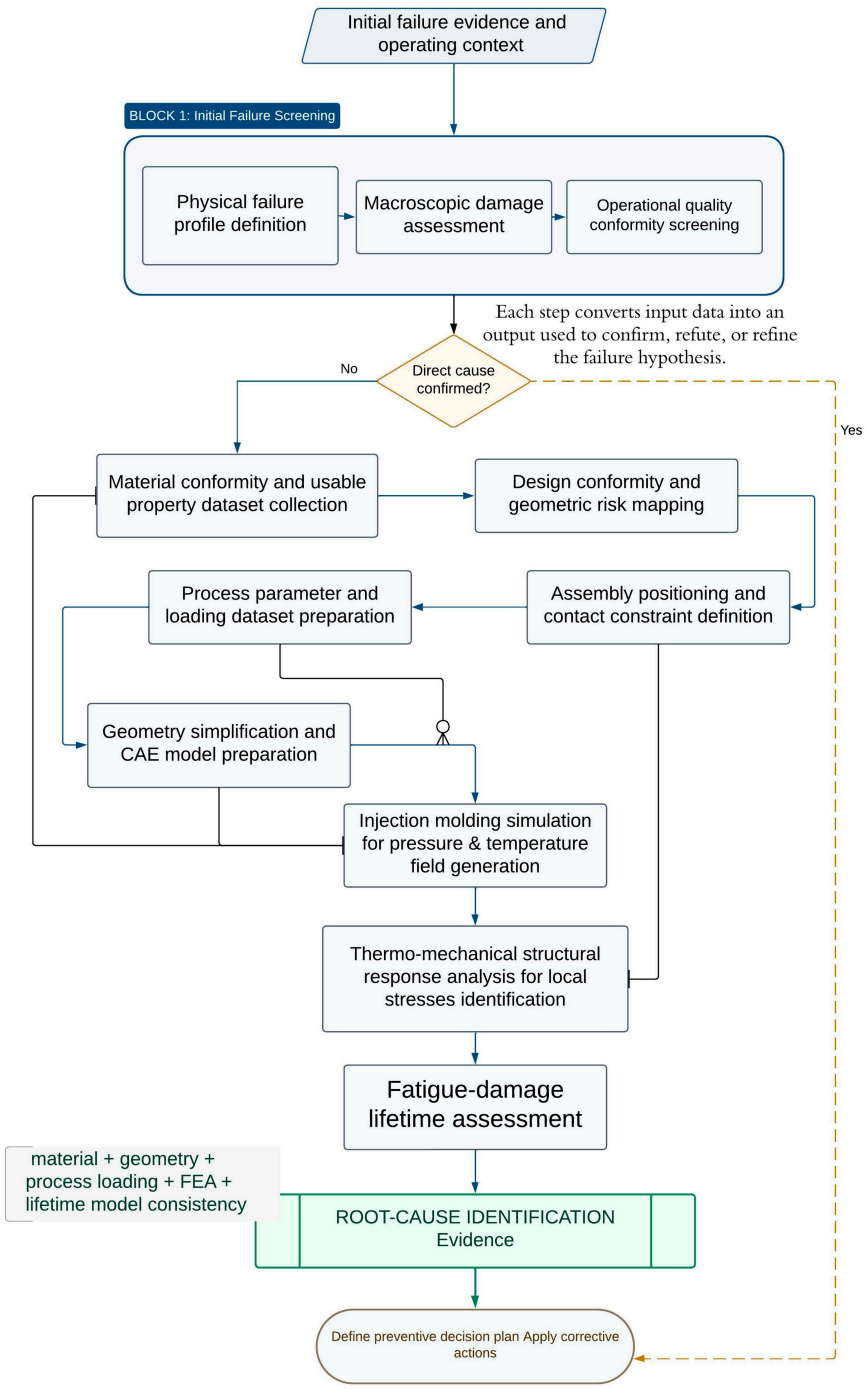
Sequential verification procedure from initial failure evidence to root-cause identification.

The investigation was conducted through a sequential verification procedure, as shown in Figure 1. If a root cause was identified at a given step, the process was stopped and an appropriate corrective action was defined.

This sequential procedure was designed to separate empirical inspection from simulation-based verification. This connection is important because numerical analysis was used only after the physical, material, geometrical, and operational evidence had been reviewed. In this way, the final root-cause conclusion was not based on simulation alone, but on a complete engineering verification chain.

The proposed methodology follows a structured root-cause analysis approach to investigate premature core insert failure in high-volume plastic injection molding. The RCA process begins with failure evidence collection, including photographs, rupture location, mold and insert references, machine data, cycle counts, and production history. Preliminary visual and operational checks are then performed, identifying possible mechanical or assembly issues. If no direct cause is found, the next step is a detailed material conformity check, assessing hardness, heat treatment, surface state, and polymer behavior. Next, geometry, tolerances, and assembly are verified by reviewing drawings and measuring critical dimensions.

Once all evidence is collected, process inputs (like injection speed, pressure, and temperature) are gathered from sensors, and the injection molding simulation is conducted [10]. Results are transferred to a structural FEA model, where local stresses are evaluated. The critical zone is identified and compared with the observed rupture location. Finally, fatigue life is estimated first by baseline LCF and then, if needed, by TMF-creep damage assessment to confirm or reject the root-cause hypothesis based on whether the observed failure matches the predicted cycles.

### 2.1. Research Scope

**Figure 2:**
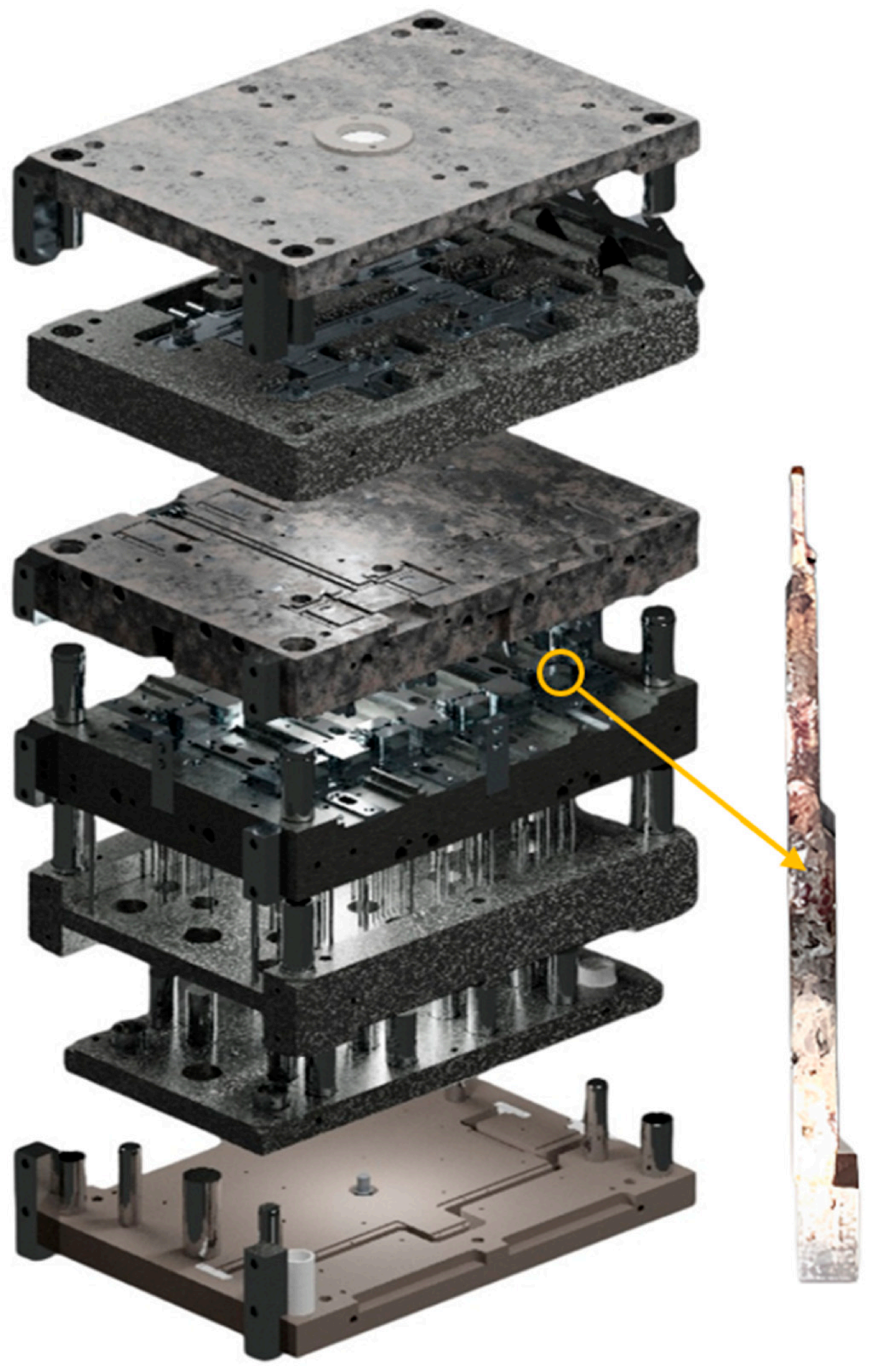
Exploded view of the injection mold assembly, highlighting the core insert’s position within the cavity.

This is a case study concerning broken core inserts inside a high-volume production plastic injection mold located in the cavity aera as shown in Figure 2.

The operating envelope of the studied injection molding case shown in Table 1 reflects production records to define the process parameters determined by the mechanical, thermal, and surrounding boundary conditions applied during each molding cycle [11].

**Figure 3:**
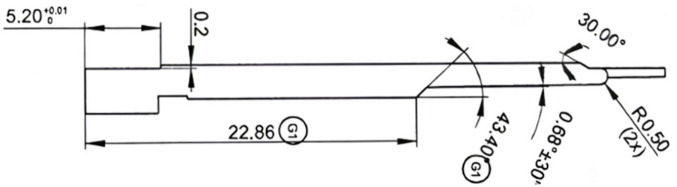
Core insert technical definition drawing.

The investigation uses samples of the broken insert, tooling and product material properties, process data, computer-aided design, and technical drawings, Figure 3.

**Figure 4:**
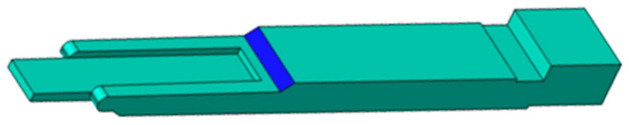
3D view of the used CAD model.

The technical drawing of the core insert provided the foundational dimensions and design intent. Based on this drawing, a 3D CAD model was created to accurately represent the geometry in three dimensions, as shown in Figure 4. This digital model serves as the basis for subsequent process and structural simulations.

Following the CAD geometry definition, the next step involved preparing the model for numerical analysis.

The investigated insert is made of **AISI H11/DIN 1.2343**, also designated as **X38CrMoV5-1**, an iron-based steel containing alloying elements that improve hardness, Table 2, strength, thermal stability, wear resistance, and fatigue resistance. For the structural finite element analysis and fatigue-damage calculations, the material is assumed to behave as homogeneous and isotropic. This assumption is adopted for numerical modeling purposes [12].

X38CrMoV5-1 is a chromium–molybdenum–vanadium hot-work tool steel, classified as a highly alloyed tool steel. These components justify its use for core inserts exposed to repeated pressure loading and transient thermal cycles in plastic injection molding. The mechanical and thermal properties of this alloy are therefore summarized in Table 3.

**Figure 5:**
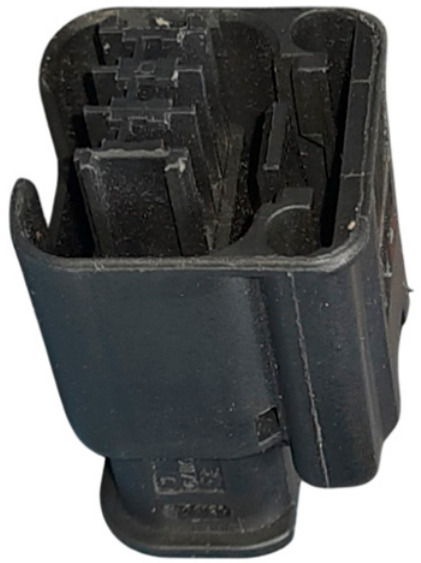
Final injected product produced using the mold.

The molded part in the studied case, Figure 5, is produced from PA6.6 GF30, a glass-fiber-reinforced polyamide containing approximately 30% glass fiber. This material is selected for applications requiring stiffness, load resistance, dimensional stability, and reduced creep compared with unfilled polyamide [13]. Therefore, its processing conditions and material properties were used as essential inputs for the injection molding simulation.

The machine used in the studied production case is the SSD Intellect 100-340 plastic injection molding machine (Sumitomo (SHI) Demag Plastics Machinery GmbH, Schwaig, Germany). Assembled with this case study mold, it generates the industrial operating environment in which the studied core insert is repeatedly exposed to pressure, thermal loading, and mold closing constraints.

### 2.2. Multiphysics Analysis Requirement

**Figure 6:**
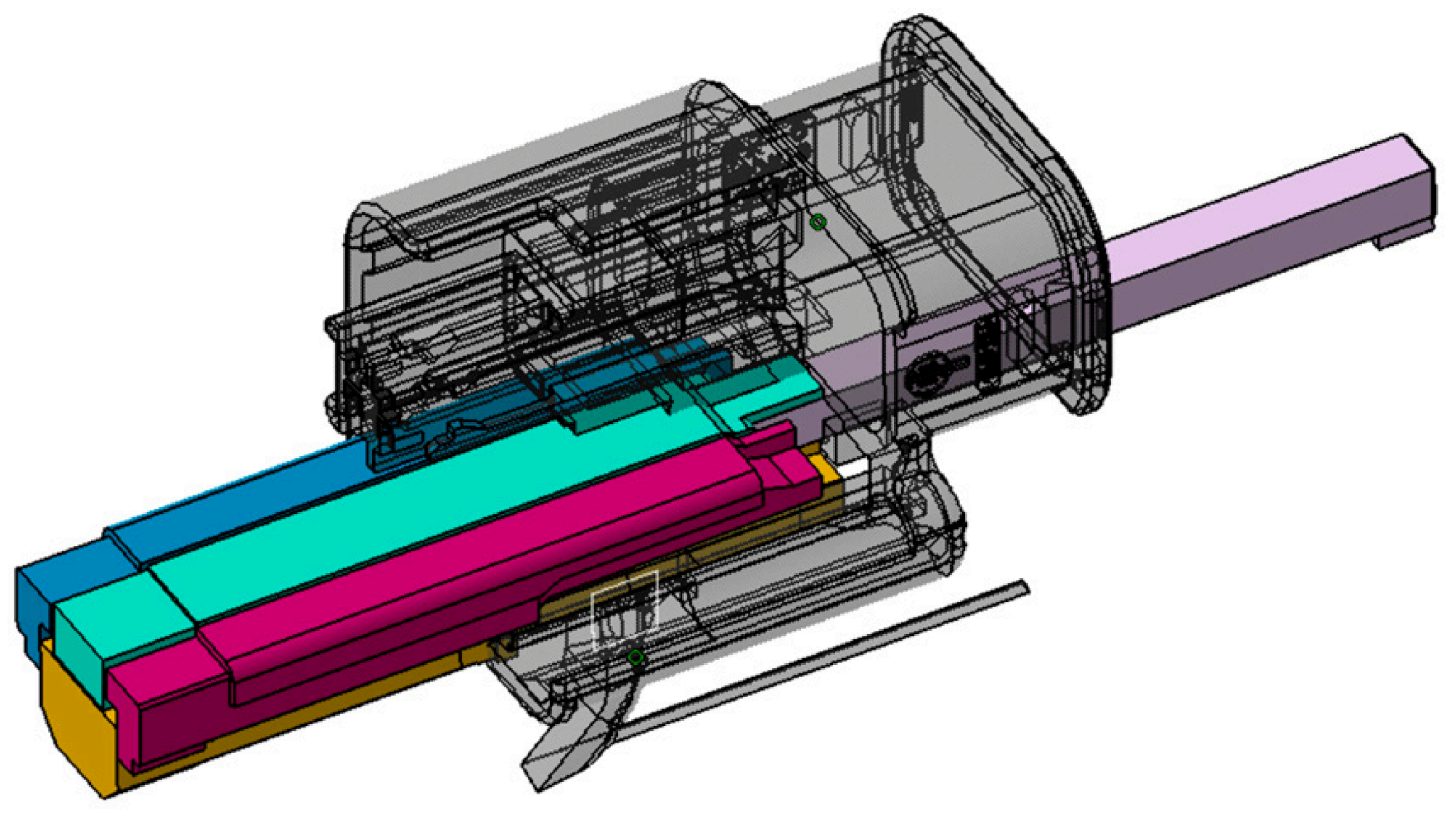
3D geometry of the insert and molded part, showing the layout and cavity components.

A 3D CAD model of the mold is required before the process-loading simulation. The model includes the cavity, the studied core insert, the surrounding mold elements, the gate region, and the contact surfaces required for the numerical analysis, Figure 6.

**Figure 7:**
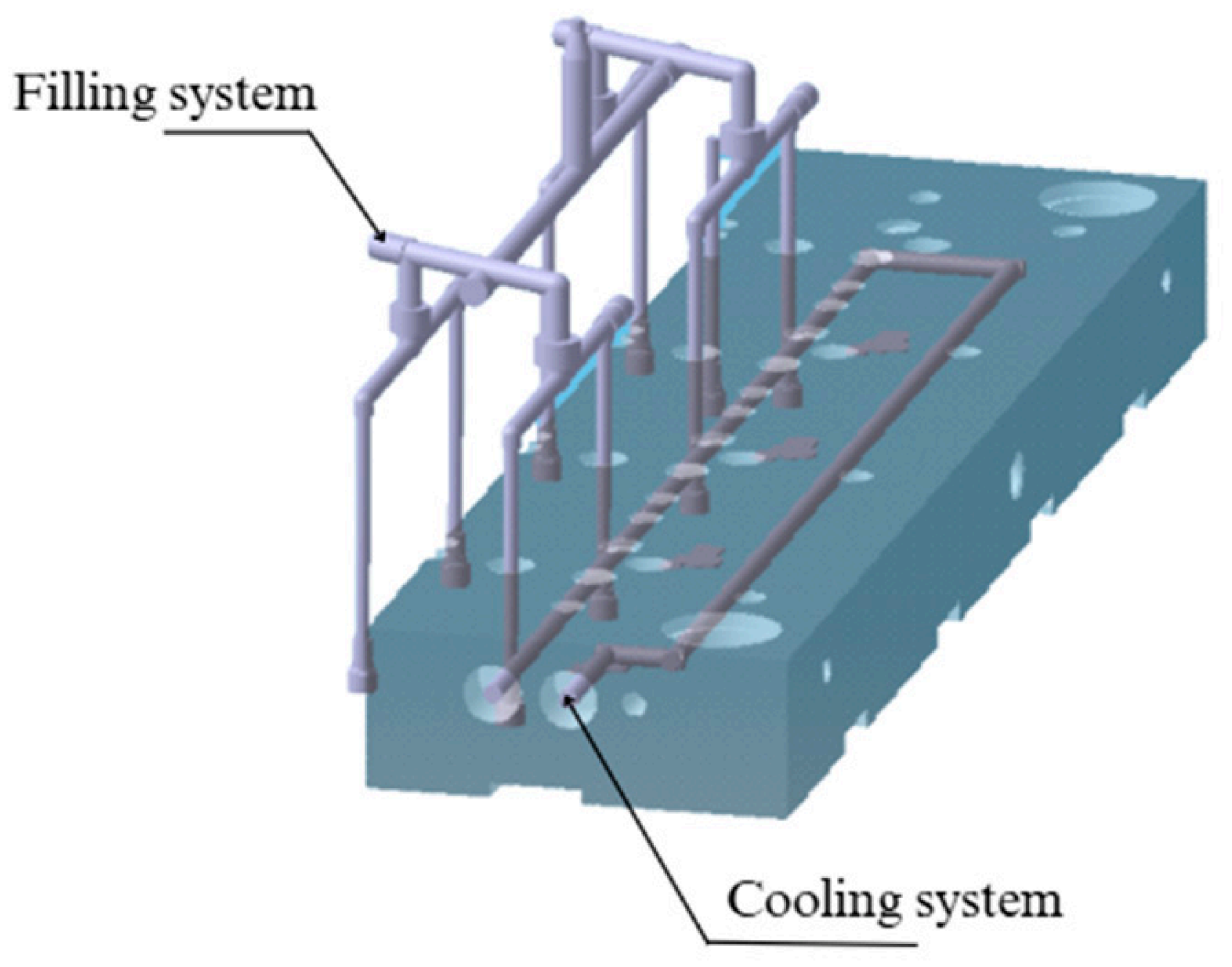
Mold system filling and cooling canalizations.

When the original CAD model is incomplete or too detailed for simulation, an optimized CAD model is prepared by preserving the geometrical features involved in the studied region and removing non-essential details for simulations, Figure 7.

**Figure 8:**
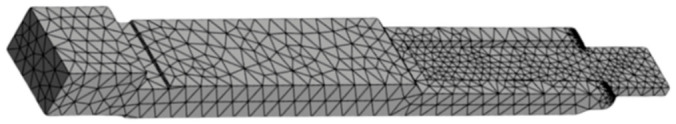
Refined finite element mesh of the core insert.

The molded polymer is defined using its material data, including flow and thermal properties required to reproduce filling, packing, and cooling behavior. The process-loading simulation is performed using the prepared cavity and insert geometry. The computational mesh is concentrated around the cavity and the studied core insert in order to maintain local accuracy while limiting computational cost as presented in Figure 8.

The 3D geometry was meshed to ensure adequate resolution in critical regions. Refinement was focused around areas where stress concentrations were anticipated, based on the insert’s geometry and where boundary conditions were evaluated.

The process inputs used in the simulation include injection pressure, injection speed, melt temperature, packing pressure, cooling time, cycle time, and gate configuration.

**Figure 9:**
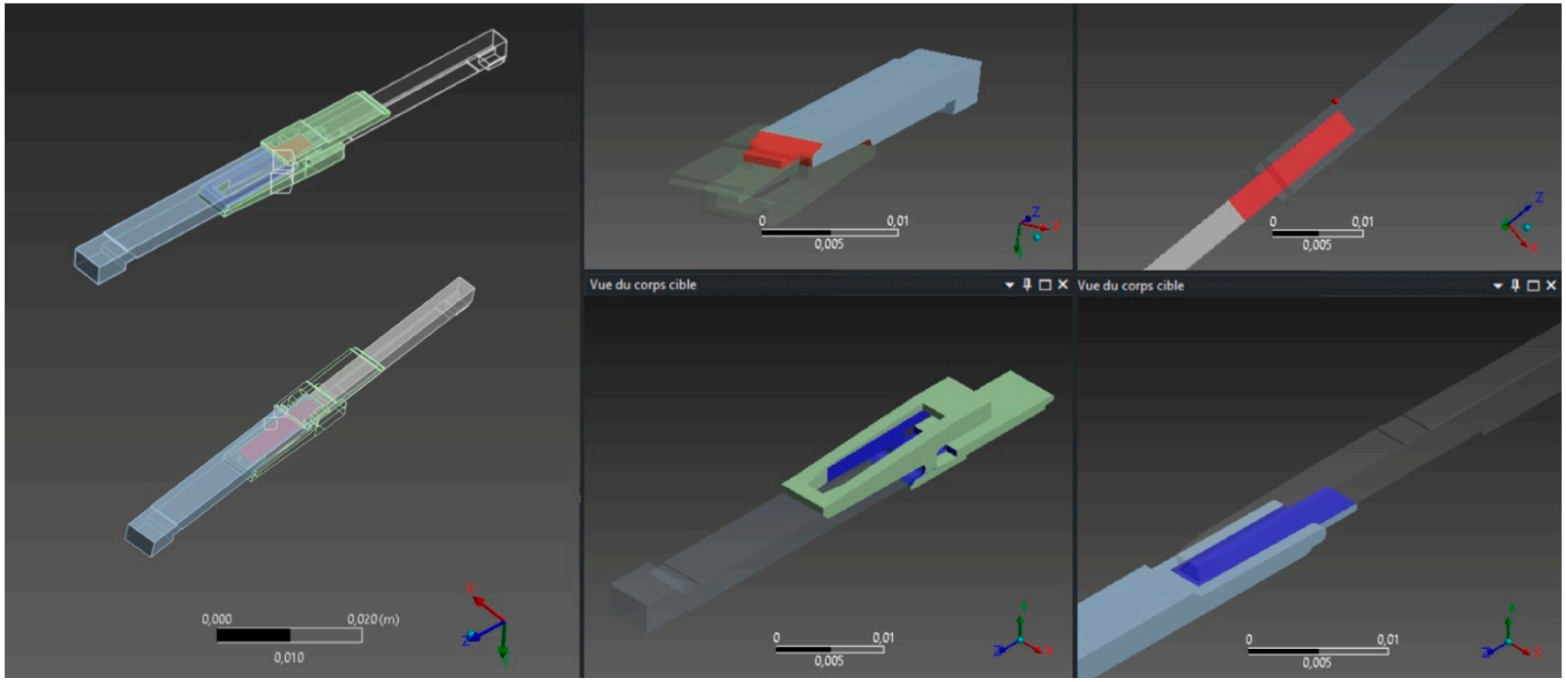
CAE model of the contact interface between the core insert and adjacent components.

Contact interfaces, support regions, and local mechanical constraints are introduced according to the mold assembly configuration, Figure 9.

The injection-molding simulation results were saved in a dedicated export folder as .cdb files. This format stores the finite element database required for the subsequent structural analysis, including the mesh, mold-base pressure field, and mold-base temperature field, while preserving their spatial correspondence for import into ANSYS 2019R3. Industrial operating data were translated into insert load inputs comprising pressure loading (injection/packing), mechanical constraints (seating/support and clamping boundary conditions), and cycle-dependent thermal loading (temperature field and gradients from repeated heating–cooling) used as inputs simulating the boundary conditions. The exported database from Moldex 3D was imported into ANSYS Mechanical APDL, where the mold-base mesh and the corresponding pressure and temperature load files were assembled within a multi-step structural analysis. Contact constraints, support conditions, and mechanical boundary conditions were then applied, after which the solution was executed and the resulting stress fields were transferred to ANSYS Workbench for post-processing and service-life evaluation.

**Figure 10:**
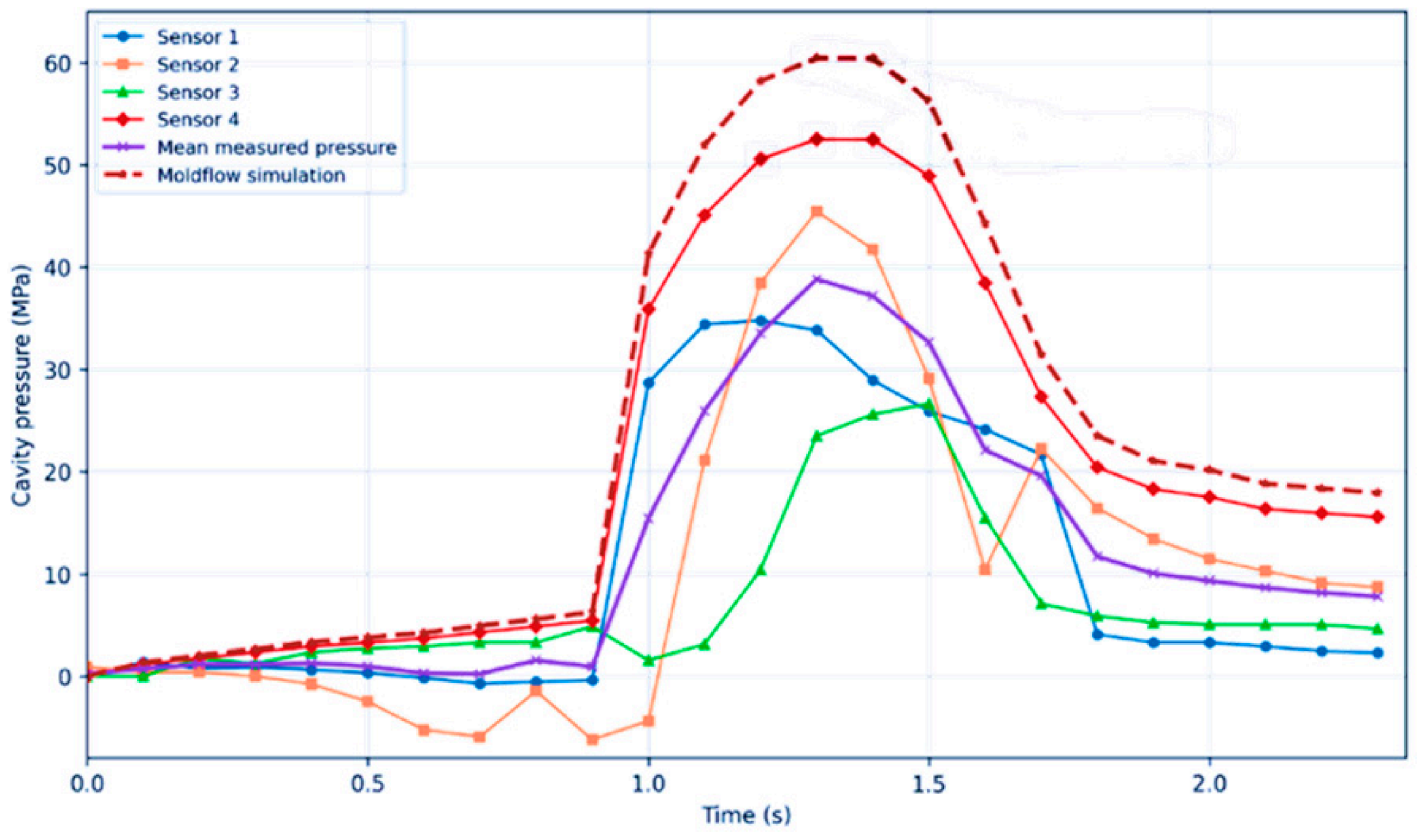
CAE and polymer pressure evolution at sensor locations.

For the process Moldflow simulation validity, the simulated pressure at the sensor location is compared with measured cavity pressure obtained using a Kistler piezoelectric cavity pressure sensor before using the results to develop the structural model analysis on the insert, Figure 10.

### 2.3. Service Life Prediction

The service-life calculation converts the local structural response into fatigue-damage indicators. The insert response is assumed homogeneous and isotropic, and the calculation is based on the FEA-extracted stress and temperature outputs.

Define the local cyclic stress state. The calculation starts with the local maximum and minimum stresses extracted from the structural model [14]. The mean and alternating stresses are defined as:

(1) $$\sigma_{a}=\frac{\left(\sigma_{max}-\sigma_{min}\right)}{2}$$

(2) $$\sigma_{m}=\frac{\left(\sigma_{max}+\sigma_{min}\right)}{2}$$

The mean stress represents the constant component of the loading cycle, while the alternating stress represents the cyclic component responsible for fatigue damage. This step converts the structural FEA stress result into fatigue-compatible input data.

The mean-stress correction to fatigue loading using the Soderberg relation [15], is used as a conservative correction where S_e_ is the endurance limit and S_y_ is the yield strength:

(3) $$\frac{\sigma_{a}}{S_{e}}+\frac{\sigma_{m}}{S_{y}}=1$$

Convert the cyclic stress–strain response. The corrected cyclic stress state is converted into cyclic strain using the Ramberg–Osgood relation [16]:

(4) $$\varepsilon =\frac{\sigma }{E}+{\left(\frac{\sigma }{K^{\prime }}\right)}^{1/n^{\prime }}$$

This step links the local stress response of the insert to its cyclic strain response using the cyclic parameters K′ and n′.

Assess baseline LCF life estimation. The baseline low-cycle fatigue life is calculated using the Coffin–Manson strain–life relation [17].

(5) $$\frac{\Delta \varepsilon }{2}=\frac{\sigma_{f}^{\prime }}{E}(2N_{f}{)}^{b}+\varepsilon_{f}^{\prime }(2N_{f}{)}^{c}$$

This equation links the cyclic strain amplitude to the number of cycles to failure by combining elastic and plastic fatigue contributions.

Formulate the coupled fatigue-creep damage model. The following equation introduces the cumulative thermo-mechanical fatigue and creep damage model.

(6) $$\omega =\omega_{\mathrm{fatigue}}+\omega_{\mathrm{creep}}$$

where: ω_fatigue_ represents damage accumulated during repeated cyclic loading, and ω_creep_ represents time-dependent damage accumulated during stress exposure at elevated temperature.

The coefficients and exponents were selected from the AISI H11 damage model, based on experimental thermo-mechanical fatigue and creep data. The fatigue contribution is then obtained from the corresponding LCF life [18]:

(7) $$\omega_{\mathrm{fatigue}}=\sum_{j=1}^{p}\frac{n_{j}}{N_{j}}\;$$

For the time-dependent creep damage formulation during stress exposure at elevated temperature. It is described using a Rabotnov-type damage law [19]:

(8) $$\frac{d\omega }{dt}\;={\left(\frac{\sigma \left(t\right)}{A_{0}}\right)}^{r}{\left(1-D_{\mathrm{creep}}\right)}^{-k}\;$$

The total scalar damage is then expressed as the sum of fatigue damage accumulated during repeated loading cycles and creep damage accumulated during stress exposure at elevated temperature:

(9) $$\omega_{\mathrm{total}}=\sum_{j=1}^{p}\frac{n_{j}}{N_{j}}+\sum_{k=1}^{q}\frac{\Delta t_{k}}{t_{r}}\leq D_{\lim}\;$$

This relation describes how damage increases with time under the combined effect of stress and temperature. In the studied insert, creep damage should not be treated as an isolated mechanism.

Predict rupture cycles using the TMF-creep model. The final service-life prediction under coupled cyclic pressure loading, transient thermal exposure, and constrained deformation is calculated as the inverse of the sum of the creep-damage increment and the thermo-mechanical fatigue damage increment accumulated during one representative molding cycle:

(10) $$N_{RP}=\frac{1}{\left(∆\omega_{c}+∆\omega_{\mathrm{TMF}}\right)}\;$$

where ∆ω_c_ represents the creep damage increment per cycle and ∆ω_TMF_ represents the thermo-mechanical fatigue damage increment per cycle, as described in the following breakdown:

(11) $$∆\omega_{c}=\frac{r+1}{A_{0}^{r}}\left(\frac{∆t\left(\sigma_{T_{max}}^{r+1}-\;\sigma_{T_{1}}^{r+1}\right)}{\left(\sigma_{T_{max}}-\;\sigma_{T_{1}}\right)\left(r+1\right)}\right)\;$$

where r is the creep stress exponent, A_0_ is the creep damage coefficient, and ∆t is the exposure time, with $\sigma_{T_{max}}$ and $\sigma_{T_{1}}$ being the stress values associated with the maximum and reference thermal states, respectively.

We have also:

(12) $$∆\omega_{\mathrm{TMF}}=\eta_{\sigma_{T}}.\psi \;$$

Since the stress-severity factor is:

(13) $$\eta_{\sigma_{T}}=\frac{\sigma_{T_{max}}-\;\sigma_{01}\left(T_{max}\right)}{\sigma_{u}\left(T_{max}\right){-\;\sigma }_{T_{max}}\;}$$

And the temperature- and thermal-gradient-corrected TMF damage intensity is:

(14) $$\psi ={\left(\frac{\sigma_{T_{max}}}{\left(1-\Phi \left(\nabla T_{max}\right)\nabla T_{max}\right)M_{0}\left(T_{max}\right)}\right)}^{\;\alpha T_{min}^{max}\beta \left(T_{max}\right)}$$

where Φ, $M_{0}\left(T_{max}\right)$, α and $\beta \left(T_{max}\right)$ are empirical coefficients describing the material resistance and damage sensitivity under cyclic thermo-mechanical loading.

This is the calculation path from FEA extracted local stress and temperature history to LCF prediction and final TMF-creep rupture-cycle estimation.

## 3. Results and Analysis

This section presents the progressive interpretation of the studied core insert failure, starting from the observable damage evidence and moving toward process simulation, structural response, and lifetime assessment. The general inspection first defines the physical failure reference, including the damaged region and probable rupture initiation zone, before these observations are correlated with the numerical results obtained from coupled process and structural analyses [20].

### 3.1. General Inspection

The fracture location, orientation, and the rupture initiation region were identified for correlation with the numerical response.

**Figure 11:**
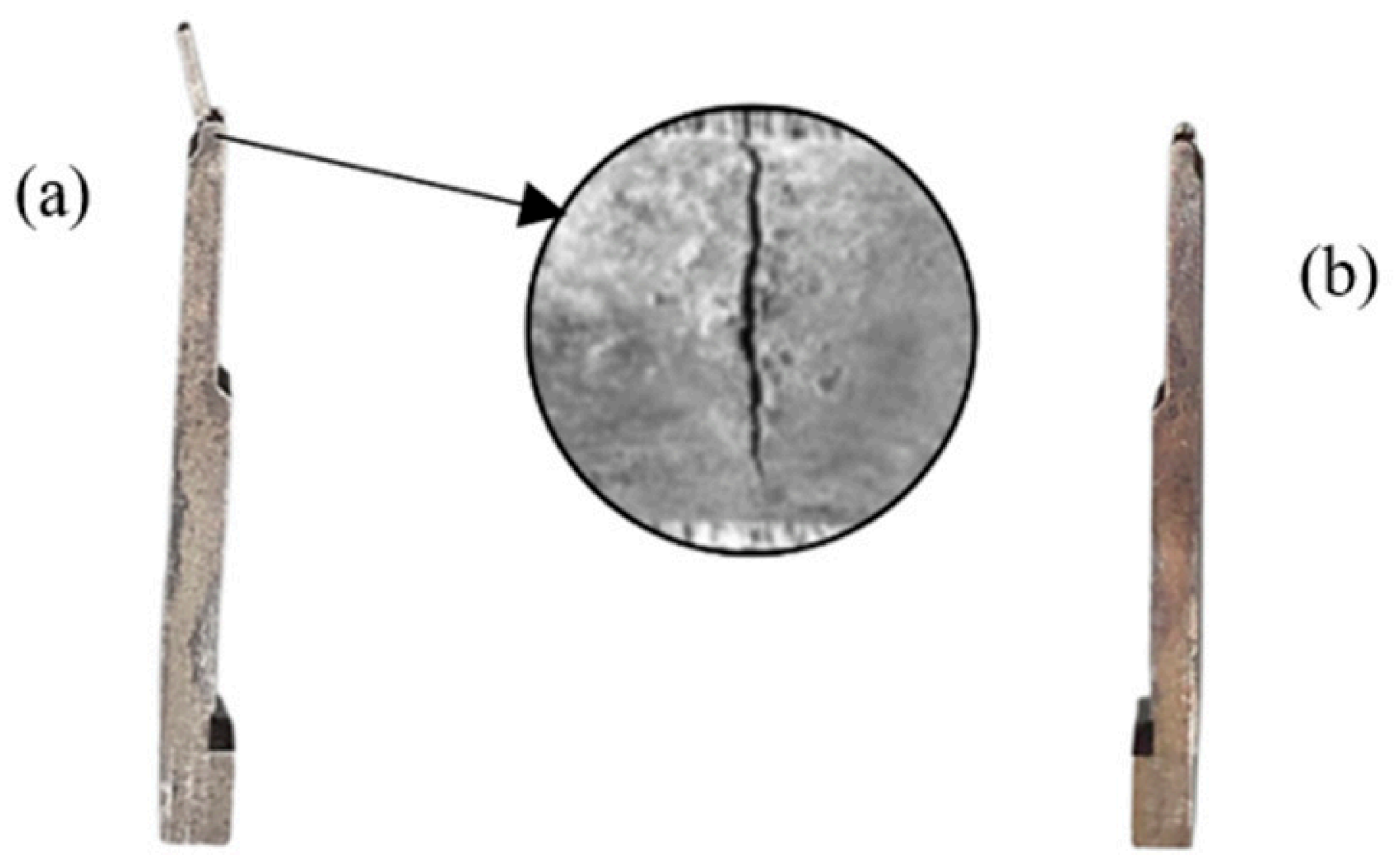
General inspection images of two failed core inserts: (**a**) partially fractured insert; (**b**) fully broken insert.

This damaged region was therefore retained as the reference zone for comparison with the critical stress region obtained from the structural finite element simulation. In Figure 11, the zoomed micrograph highlights the rupture initiation site under magnification.

Although the damaged region can be visually retained as the reference rupture zone, the raw observation alone does not allow the crack initiation mechanism to be definitively identified. The micrograph indicates the probable rupture initiation site, but it does not by itself explain how the crack started through:

- Mechanical overload in regions of excessive local stress concentration;
- Thermal fatigue caused by repeated heating and cooling cycles;
- Contact-induced constraint generated by insert positioning, support conditions, and mold assembly interaction;
- Process-induced loading caused by repeated injection pressure, holding pressure, and transient thermal exposure;
- Progressive thermo-mechanical damage resulting from the interaction between cyclic pressure loading, thermal gradients, and constrained insert deformation.

### 3.2. Process Simulation Results

The injection molding process simulation provided the two main process-derived fields required for the structural analysis: the transient temperature field and the polymer pressure field. These results describe how the molded polymer thermally and mechanically loads the cavity, the core insert, and the surrounding mold structure during the molding cycle. They therefore define the multiphysics boundary conditions transferred to the structural FEA model.

#### 3.2.1. Heat Distribution in the Mold Cavity

**Figure 12:**
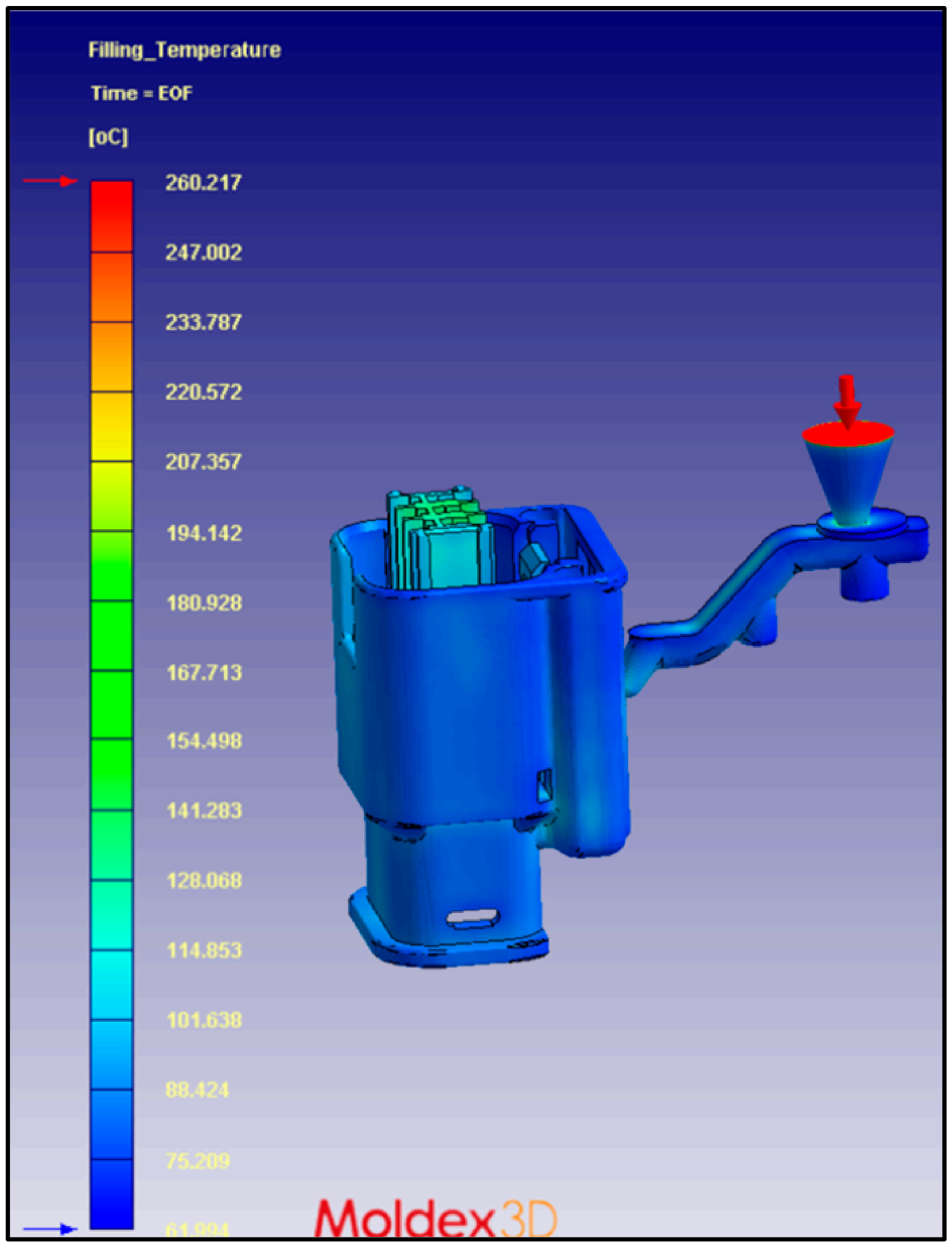
Thermal field simulation results of temperature distribution in the cavity.

The simulated heat distribution shows the transient thermal exchange between the hot polymer, the cavity surface, the core insert, and the mold plate, Figure 12. During filling, the polymer introduces heat into the cavity, while cooling progressively extracts this heat through the metallic mold structure.

This produces a non-uniform temperature field and a thermal gradient between the polymer core, the cavity wall, and the surrounding mold components. This result is important because the resulting thermal gradient contributes to thermal strain and local thermo-mechanical loading in the insert.

#### 3.2.2. Polymer Pressure Applied to the Cavity Surface

**Figure 13:**
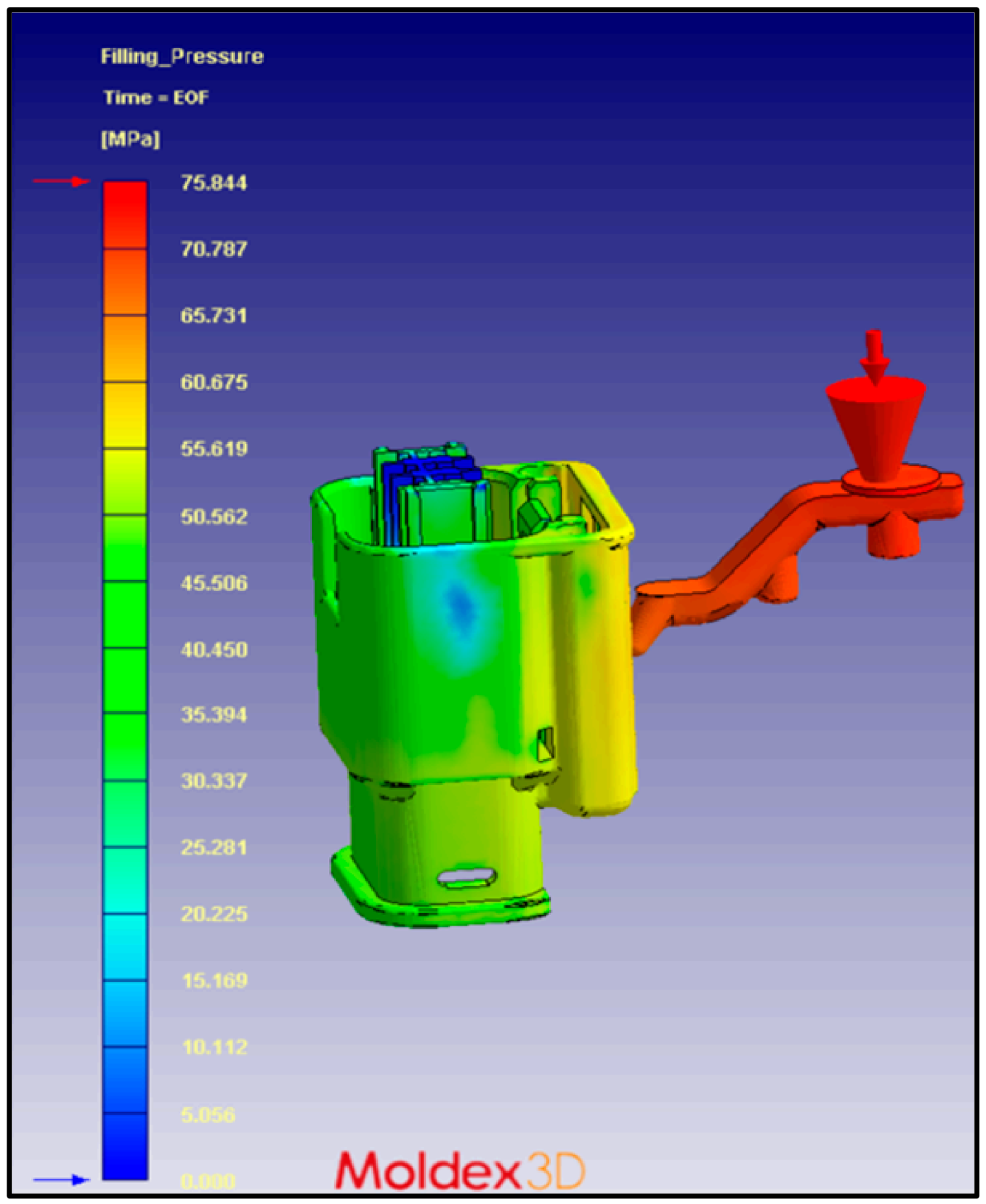
Simulated polymer pressure distribution within the mold cavity during the packing/holding stage.

The simulated pressure field shows the mechanical loading applied by the polymer on the cavity and insert surfaces during filling and holding, Figure 13. During filling, the polymer pressure evolves according to the flow path and local cavity resistance. After filling, holding pressure is maintained to compensate for polymer shrinkage during cooling and solidification.

The selected process state corresponds to the early packing/holding stage, after cavity filling and before complete cooling. At this stage, the polymer remains highly pressurized to compensate for shrinkage, while the molded part is still hot and transfers heat toward the cavity wall, core insert, and mold plate. This stage therefore represents a critical coupled thermo-mechanical loading condition, in which polymer pressure and thermal gradients act simultaneously on the insert surfaces.

This process state was retained as the critical loading condition for transferring the maximum thermal and mechanical boundary conditions to the structural FEA model.

### 3.3. Process Structural Response Assessment and Numerical Results

Before meshing, the geometries of the molded part, cavity, core insert, and adjacent mold components were prepared as simulation-ready three-dimensional models. Boolean operations, including union, subtraction, intersection, and volume extraction, were used to reconstruct the polymer volume and define the contact interfaces between the molded part and the tooling components. When the original CAD geometry was incomplete or unsuitable for direct simulation, reverse-engineering tools were employed to reconstruct missing surfaces, repair discontinuities, close gaps, and improve geometric consistency. Small features that did not influence polymer flow, heat transfer, or the local structural response were removed to improve mesh quality and reduce computational cost. However, critical features such as the insert profile, rupture-sensitive regions, contact surfaces, wall thickness variations, and flow restrictions were preserved to maintain the physical accuracy of the injection molding and structural simulations.

**Figure 14:**
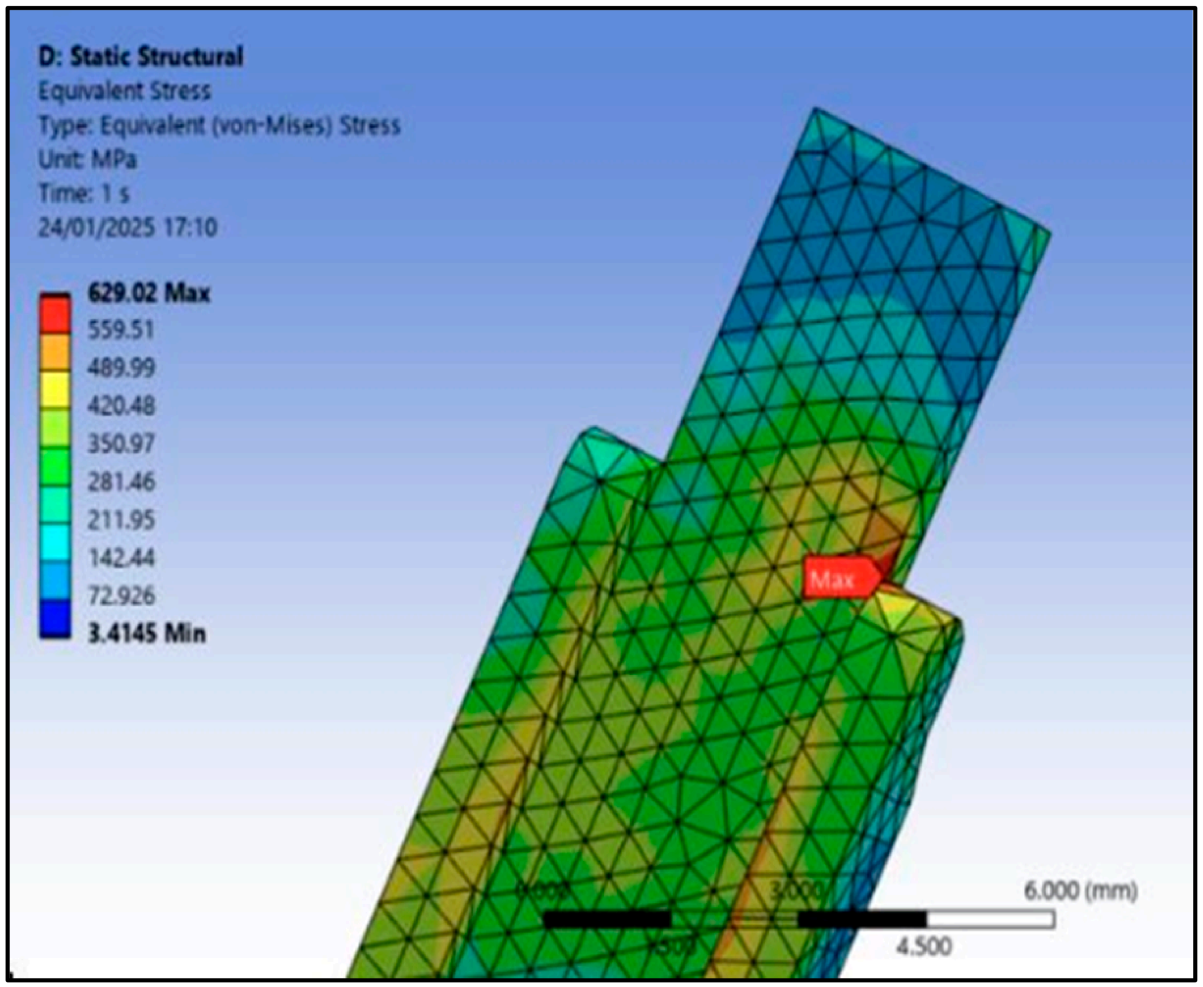
Simulated numerical results of the structural finite element analysis.

The structural analysis was performed using the maximum thermal and mechanical boundary conditions extracted from the injection molding process simulation. The transferred polymer pressure and temperature fields were applied to the AISI H11 core insert model to evaluate the local stress response under realistic operating conditions, Figure 14. The objective of this step was to identify the critical stress concentration region and associate it with the observed rupture initiation zone.

The structural finite element analysis showed a localized von Mises stress of approximately 629 MPa at the rupture-initiation region. This value was retained as the local mechanical response of the insert and used as the stress input for the subsequent fatigue-damage calculation.

**Figure 15:**
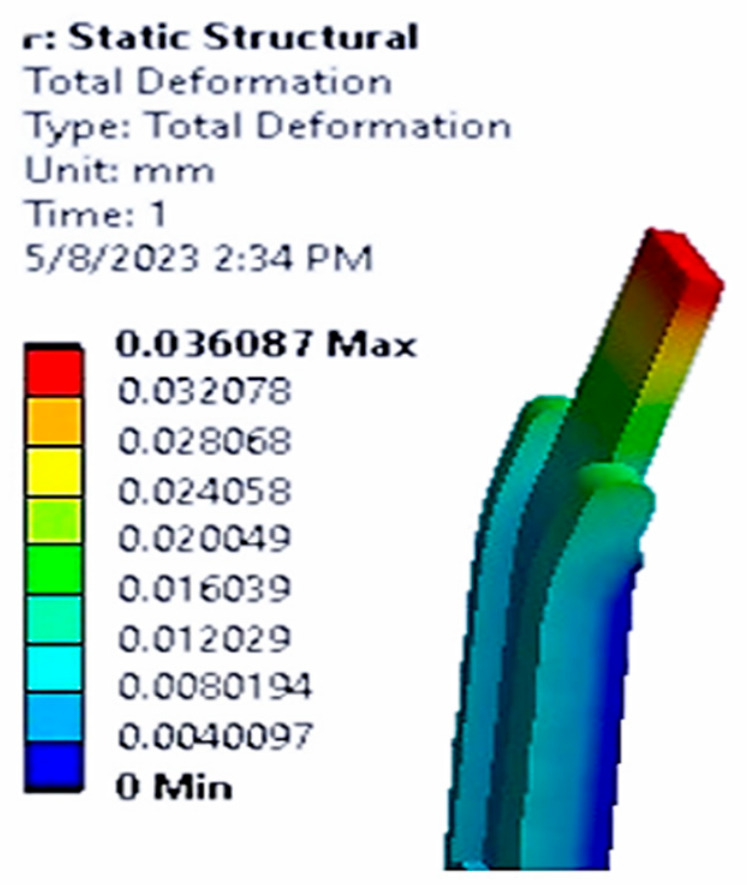
Total deformation results.

The numerical model also reported a thermally induced displacement of approximately 0.03 mm in constrained regions, indicating the local deformation response of the insert under coupled pressure and thermal loading, Figure 15. These values were retained for the service-life calculation. The stress, strain, and temperature history extracted from this region were used as input for the baseline LCF and TMF-creep damage calculations.

The calculation using the low-cycle fatigue model predicted: $N_{f}=\;20,000\;cycles$. The thermo-mechanical fatigue and creep model predicted: $N_{RP}=\;7043.639\;cycles$.

For our case, with the molding cycle time of 15.2 s, in the studied production scope, the number of life cycles it corresponds to a functional production time of $t_{FP}=29.7\;h$.

### 3.4. Static Critical Stress Assessment

Using CAE tools, the structural analysis reported a localized von Mises stress of approximately 629 MPa at the rupture-initiation region. This stress level is below the static strength level of the hardened AISI H11 condition used in the calculation. Therefore, the stress value is not retained as evidence of single-cycle static overload. Its role is to define the local stress state used for fatigue-damage assessment.

The numerical simulation model also reported a thermally induced displacement of approximately 0.03 mm in constrained regions. This displacement should be considered together with assembly dimensional tolerances and contact conditions. Although limited in magnitude, it may become relevant when the insert operates with small clearances, tight fitting surfaces, or local contact zones close to tolerance limits.

The baseline LCF prediction indicates that mechanical fatigue alone is insufficient and that the analysis must proceed toward the following step.

### 3.5. TMF-Creep Damage Assessment of the Failure Root-Cause Hypothesis

The observed annual breakage frequency of 321 cases corresponds to the studied core insert, counted per cavity and per mold across similar molds producing the same product, for an average service life of approximately 27.3 production hours before breakage under a continuous mass manufacturing environment. This value is close to the TMF-creep predicted lifetime of 29.7 h, supporting the consistency between the model prediction and the real production records.

The TMF-creep model uses the same local stress-temperature values extracted from the rupture-initiation region. This model evaluates the combined effect of cyclic stress, transient thermal exposure, and time-dependent damage during repeated molding cycles.

## 4. Discussion

### 4.1. From Observed Failure to Evidence-Based Verification

The analysis was not limited to identifying a cracked insert or reporting a high local stress value. It followed a verification logic in which each possible explanation was checked against the available evidence. The visual inspection first defined the rupture location, crack orientation, and probable initiation region. These observations gave the physical reference needed to compare the failed part with the numerical response. The process simulation then supplied the pressure and temperature fields, while the structural model converted these fields into local stress and displacement responses. This sequence is important because the failure of a core insert cannot be evaluated only from machine settings or from the visual crack shape; it must be linked to the local response of the insert as a key element within the mold assembly system.

### 4.2. Verification of Non-Retained Failure Factors

Before retaining the final damage mechanism, the first estimated failure factors were checked against the available evidence. The maximum von Mises stress of approximately 629 MPa remains below the static strength level of the hardened AISI H11 condition used in the calculation; therefore, single-cycle static overload is not retained. The global clamping force is also not retained as the main cause because the available machine clamping force exceeds the calculated minimum requirement. Similarly, the thermally induced displacement of approximately 0.03 mm does not confirm assembly tolerance excess as a direct root cause, but it highlights improvement areas related to tolerance control, insert seating, contact-surface quality, and local support conditions.

The baseline low-cycle fatigue prediction was then used as a mechanical fatigue verification step. The model predicted approximately 20,000 cycles, while the observed failure occurred near 7000 cycles. This mismatch shows that baseline mechanical fatigue alone is not sufficient to explain the studied failure. Therefore, static overload, insufficient clamping force, assembly/tolerance effects, and LCF prediction model are treated as non-retained failure factors. Their verification supports the need to proceed toward the coupled thermo-mechanical fatigue and creep assessment.

### 4.3. Interpretation of Key Findings

The main value of the results lies in the progressive verification of the failure mechanism. Visual inspection alone identifies the damaged region, but it cannot explain the failure mechanism. Structural simulation adds the local mechanical response, while lifetime modeling checks whether this response can reproduce the observed failure range. This sequence transforms the analysis from simple failure observation into evidence-based root-cause verification.

The comparison between the baseline fatigue model and the coupled TMF-creep model also clarifies the limitation of isolated approaches. Static stress checking locates whether immediate overload is possible, and LCF provides a mechanical fatigue reference, but neither is sufficient alone for the studied case. The added value of the final formulation is that it introduces the missing non-isothermal and time-dependent damage components required to represent high-volume injection molding conditions.

From an industrial perspective, the findings show why insert breakage should be assessed through a connected process-to-structure-to-damage workflow. The objective is not only to calculate a lifetime after failure, but to identify which data must be monitored during production: pressure history, thermal exposure, cycle time, cooling conditions, contact constraints, maintenance events, and failure frequency. This creates the basis for automating tooling-integrity assessment and moving from corrective replacement toward predictive monitoring of mold components.

### 4.4. Improvement Potential and Automation for High-Volume Production Usage

For high-volume production, the main perspective is to move from post-failure investigation toward continuous tooling-integrity monitoring. This requires systematic tracking of the variables that define the molding environment, including cycle time, injection pressure, holding pressure, temperature history, cooling conditions, clamping data, maintenance operations, insert replacements, and failure records. Once recorded, these data can be connected to the CAE-based workflow to update the estimated damage state of the insert during production, instead of waiting for rupture to occur.

Future work should automate the process-to-structure-to-damage sequence. In an automated implementation, production data would feed the process-loading model, pressure and temperature fields would update the structural response, and the extracted local stress–temperature history would update the damage indicator or remaining-life estimate. With sufficient historical cases, machine-learning models could be added as a diagnostic layer to recognize abnormal loading patterns, classify probable failure mechanisms, and recommend preventive actions [21]. This would transform the framework from a case-specific failure analysis tool into a decision-support system for predictive maintenance and tooling reliability.

The same logic can be extended beyond core inserts to other functional mold components. Mold cavity elements, sliders, and support regions can be treated as active thermo-mechanical parts exposed to polymer pressure, heat transfer, contact constraints, clearance variation, and repeated cycling. Future investigations should therefore include contact evolution, dimensional tolerance effects, local constraint changes, thermal expansion, and progressive damage accumulation across different mold components. This extension would strengthen the framework as a broader mold reliability assessment method for high-volume injection molding.

## 5. Conclusions

This study demonstrates a reproducible root-cause identification approach for an industrial AISI H11 core insert failure under high-volume injection molding conditions. The main contribution is the integration of production evidence, process-derived thermo-mechanical loading, structural finite element response, and fatigue-damage modeling into a single root-cause assessment sequence. The results show that, in this specific case, failure interpretation cannot be adequately performed using visual inspection, static stress evaluation, or low-cycle fatigue-life estimation alone. Visual inspection defined the probable rupture-initiation region, while FEA evaluated the local stress response in the studied area under the surrounding boundary conditions. The localized von Mises stress was below the static strength critical value for the AISI H11 material, whereas the baseline LCF prediction overestimated the observed service life.

The final prediction of approximately 29.7 h under the studied cycle time and the thermo-mechanical fatigue and creep mechanism is a plausible and numerically consistent hypothesis. Therefore, a progressive damage process localized at the critical stress region governed the studied premature breakage, providing a practical basis for failure-risk evaluation in high-volume injection molding tooling. Within the limits of the investigated case, this approach supports the transition from isolated post-failure checks toward service-life assessment, real-time monitoring, improved predictive maintenance planning, and more accurate preventive actions to reduce core insert failure incidents.

## Figures and Tables

**Table 1:** The experimental operating envelope of the studied case.

Molding Process Parameter	Reference Value	
Machine and cycle settings	Injection speed = 120 mm/s	Injection time = 0.67 s
	switch-over = 20.00 ccm/1180 bar	holding pressure = 700 bar for 2.5 s
	cooling time = 7.00 s	cycle time = 15.2 s
Global mold closure	Machine clamping force = 820 kN;	Locally applies 1 kN of contact force insert-level
	minimum requirement = 660 kN.	
Temperature-control inputs	Injection unit/nozzle 250 °C	feed cooling 60 °C
	feed transition 245 °C	Slides 54–63 °C
	hot-runner/feed path 240–290 °C	T_min_ = 22 °C; T_max_ = 297 °C.

**Table 2:** Chemical composition of H11/X38CrMoV5-1.

C	Si	Cr	Mo	V	Mn
0.38	1.10	5.00	1.30	0.40	0.40

**Table 3:** Mechanical and thermal material properties.

Material Property	Value (For H11)
Density	7.80 g/cc
Hardness, Rockwell C	55
Tensile Strength, Ultimate	1990 MPa
Tensile Strength, Yield	1650 MPa
Elongation at Break	9.0%
Modulus of Elasticity	210 GPa
Poisson’s Ratio	0.30
Shear Modulus	81 GPa
Specific Heat Capacity	0.460 J/g-°C
Thermal Conductivity	24.6 W/m-K

## Data Availability

The raw data supporting the conclusions of this article will be made available by the authors on request.

## Abbreviations

The following abbreviations are used in this manuscript:

CAE	Computer-Aided Engineering	
BC	Boundary Conditions	
FEA	Finite Element Analysis	
HRC	Rockwell hardness scale C	
PA	Polyamide	
CAD	Computer-Aided Design	
N_f_	Number of cycles to failure from the LCF model	
N_RP_	Predicted rupture cycles from the TMF-creep model	
RCA	Root-Cause analysis	
t_FP_	Functional production time	
**Symbols**		
**Parameter Symbol**	**Designation**	**unit**
E	Young’s modulus	Gpa
K′	Cyclic strength coefficient	Mpa
n′	Cyclic strain-hardening exponent	—
σ′_f_	Fatigue strength stress coefficient	MPa
ε′_f_	Fatigue strain coefficient	—
b	fatigue strength exponent	—
c	fatigue ductility exponent	—
A_0_	Creep stress coefficient	MPa
r	Creep stress exponent	—
k	Creep damage evolution exponent	—
T_max_	Maximum process temperature	°C
D_Lim_	Normalized critical damage limit	—
M_0_ (T_max_)	Thermal resistance parameter	MPa
α	TMF damage sensitivity coefficient	—
β (T_max_)	Thermal damage exponent	—
t_c_	Insert’s service life	h
t_FP_	Functional production time	h
